# Supplementary material for: Molecular detection of SARS-CoV-2 using a reagent-free approach
Source: PLoS One. 2020 Dec 7;15(12):e0243266. doi: 10.1371/journal.pone.0243266 (PMC7721139; doi:10.1371/journal.pone.0243266)
Supplement: S1 Table — (DOCX) [file pone.0243266.s001.docx]

**S1 Table.** Sequences of primers and probes for SARS-CoV-2 (N1), AoGV and PVY and optimised final concentrations for their use in real-time RT-PCR.

| **Oligo Name** | **Sequence (5'>3')** | **Final concentration** |
| --- | --- | --- |
| 2019-nCoV_N1-F | GACCCCAAAATCAGCGAAAT | 500 nM |
| 2019-nCoV_N1-R | TCTGGTTACTGCCAGTTGAATCTG | 500 nM |
| 2019-nCoV_N1-Pr | 6FAM- ACCCCGCATTACGTTTGGTGGACC--BHQ1 | 150 nM |
| AoGV-F | CCTATTTCCATCGCCCACTA | 500 nM |
| AoGV-R | GCGACACCTCAATCATAATCTC | 500 nM |
| AoGV-Pb | YAK-AGTCTATATCGGTACCACGTCTGCC-BHQ1 | 150 nM |
| PVY-F | CCAAACCCGAACAAAGGAAAG | 133 nM |
| PVY-R | GGGCATTCTCATTTTGGACG | 133 nM |
| PVY-Pb | HEX-TGCAGGCACATCTGGGACACATAC-BHQ1 | 50 nM |
| PVY Ultramer® RNA Oligo | AGCATCCAGCCAAACCCGAACAAAGGAAAGGATAAGGAT GTTAATGCAGGCACATCTGGGACACATACTGTGCCGAGA ATCAAGGCTATCACGTCCAAAATGAGAATGCCCACAAGC | ≈ 10^7^ copies/mL |
