# Supplementary material for: Molecular detection of SARS-CoV-2 using a reagent-free approach
Source: PLoS One. 2020 Dec 7;15(12):e0243266. doi: 10.1371/journal.pone.0243266 (PMC7721139; doi:10.1371/journal.pone.0243266)
Supplement: S2 Table — (DOCX) [file pone.0243266.s002.docx]

**S2 Table.** RT-PCR mix preparation. Left: Reagent volumes required to prepare the RT-PCR mix used for the detection of SARS-CoV-2 in the reference assay (NA/ABI) or following heat treatment (Meridian Fast 1-Step). Right: Reagent volumes required for the preparation of the duplex SARS-CoV-2/PVY mix. NA: Nucleic acid extraction. HT: Heat Treatment.

| **ABI TaqManFast Virus (NA/ABI)** | |  | **Meridian Fast 1-Step RT-PCR (HT/Duplex)** | |
| --- | --- | --- | --- | --- |
| Reagent | Volume (μL) |  | Reagent | Volume (μL) |
| Fast Virus 1-Step Mix (4X) | 12.5 |  | Fast 1-Step Mix (2X) | 25 |
| Primer (10μM) | 2.5 |  | Primer (10μM) | 2.5 |
| Probe (3μM) | 2.5 |  | Probe (3μM) | 2.5 |
| Water | 12.5 |  | PVY Primer (10μM) | 0.83 |
| Template | 20 |  | PVY Probe (3μM) | 0.83 |
|  |  |  | PVY RNA (10^9^/mL) | 0.83 |
|  |  |  | MMLV-RT | 0.5 |
| **Meridian Fast 1-Step RT-PCR (HT/Fast 1-Step)** | |  | RNase Inhibitors | 1 |
| Reagent | Volume (μL) |  | Template | 20 |
| Fast 1-Step Mix (2X) | 25 |  |  |  |
| Primer (10μM) | 2.5 |  |  |  |
| Probe (3μM) | 2.5 |  |  |  |
| MMLV-RT | 0.5 |  |  |  |
| RNase Inhibitors | 1 |  |  |  |
| Template | 20 |  |  |  |

^1^ AoGV detection was performed using the HT/Fast 1-Step prepared in 10μL final volume reactions with extracted AoGV added to 2×10^5^ genomes/mL.
