# Supplementary material for: Molecular detection of SARS-CoV-2 using a reagent-free approach
Source: PLoS One. 2020 Dec 7;15(12):e0243266. doi: 10.1371/journal.pone.0243266 (PMC7721139; doi:10.1371/journal.pone.0243266)
Supplement: S3 Table — (DOCX) [file pone.0243266.s003.docx]

**S3 Table**. Detection of the AoGV and PVY internal controls in multiple parallel diagnostics runs using non-screened samples (n=265). Ct values recorded using either a DNA (AoGV) or an RNA (PVY) internal control were recorded for each sample. The proportion of samples requiring repeat testing (extraction) due to IC failure (no amplification or delayed amplification) was calculated in each case. Blue: samples displaying PVY inhibition due to the presence of a high viral load of SARS-CoV-2 (polymerase out-competition). Yellow: samples showing inhibition in both the AoGV and the PVY assays (PCR inhibition). Green: samples showing PVY-only inhibition (RT inhibition). Orange: samples displaying delayed amplification of the internal control (4 cycles behind the water control). Grey: samples displaying a high background fluorescence leading to inaccurate Ct value estimation. ND: Not detected.

| **Patient num** | **AoGV (Ct value)** | **PVY (Ct value)** |  |  |  |  |
| --- | --- | --- | --- | --- | --- | --- |
| 20000 | 34.1 | 29.5 |  |  | PVY -ve, SARS-CoV-2 strong positive | |
| 20001 | 34.2 | 29.3 |  |  | PCR inhibitor |  |
| 20002 | 34.5 | 29.4 |  |  | RT inhibitor |  |
| 20003 | 34.0 | 29.7 |  |  | late Ct |  |
| 20004 | 34.1 | 29.7 |  |  | High Background |  |
| 20005 | 34.2 | 31.0 |  |  |  |  |
| 20006 | 34.3 | 29.3 |  |  |  |  |
| 20007 | 34.4 | 29.7 |  |  | **AoGV** | **PVY** |
| 20008 | 34.1 | 29.7 |  | Total | 265 | 265 |
| 20009 | 34.4 | 29.8 |  | Fail | 3 | 7 |
| 20010 | 35.2 | ND |  | Late | 0 | 2 |
| 20011 | 33.8 | 30.9 |  | Repeat rate | 1.13% | 3.40% |
| 20012 | 34.3 | 30.0 |  |  |  |  |
| 20013 | 34.1 | 30.0 |  |  |  |  |
| 20014 | 33.9 | 30.0 |  | **n=265** |  |  |
| 20015 | 34.1 | 31.3 |  |  |  |  |
| 20016 | 34.3 | 29.8 |  |  |  |  |
| 20017 | 29.2 | 35.0 |  |  |  |  |
| 20018 | 33.0 | 29.2 |  |  |  |  |
| 20019 | 34.5 | 32.8 |  |  |  |  |
| 20020 | 34.2 | 30.5 |  |  |  |  |
| 20021 | 34.6 | 32.2 |  |  |  |  |
| 20022 | 34.4 | 29.9 |  |  |  |  |
| 20023 | 34.0 | 30.0 |  |  |  |  |
| 20024 | 34.4 | 29.9 |  |  |  |  |
| 20025 | 34.3 | 31.8 |  |  |  |  |
| 20026 | 34.3 | 29.9 |  |  |  |  |
| 20027 | 34.6 | 29.6 |  |  |  |  |
| 20028 | 34.5 | 29.7 |  |  |  |  |
| 20029 | 34.1 | 30.0 |  |  |  |  |
| 20030 | 34.4 | 34.7 |  |  |  |  |
| 20031 | 34.3 | 29.7 |  |  |  |  |
| 20032 | 32.8 | 29.6 |  |  |  |  |
| 20033 | 18.3 | 28.7 |  |  |  |  |
| 20034 | 34.2 | 30.0 |  |  |  |  |
| 20035 | 34.1 | 29.6 |  |  |  |  |
| 20036 | 34.0 | 27.7 |  |  |  |  |
| 20037 | 35.2 | ND |  |  |  |  |
| 20038 | 29.0 | 29.3 |  |  |  |  |
| 20039 | 34.3 | 31.1 |  |  |  |  |
| 20040 | 34.0 | 30.3 |  |  |  |  |
| 20041 | 34.2 | 29.9 |  |  |  |  |
| 20042 | 34.6 | 29.5 |  |  |  |  |
| 20043 | 34.0 | 30.6 |  |  |  |  |
| 20044 | 34.3 | 30.0 |  |  |  |  |
| 20045 | 33.8 | 29.9 |  |  |  |  |
| 20046 | 34.7 | 29.8 |  |  |  |  |
| 20047 | 34.0 | 29.7 |  |  |  |  |
| 20048 | 34.3 | 30.1 |  |  |  |  |
| 20049 | 27.6 | 29.9 |  |  |  |  |
| 20050 | 34.1 | 29.9 |  |  |  |  |
| 20051 | 33.7 | 27.6 |  |  |  |  |
| 20052 | 34.1 | 29.9 |  |  |  |  |
| 20053 | 34.5 | 30.0 |  |  |  |  |
| 20054 | 34.1 | 30.1 |  |  |  |  |
| 20055 | 34.2 | 29.9 |  |  |  |  |
| 20056 | 33.8 | 30.0 |  |  |  |  |
| 20057 | 34.2 | 29.6 |  |  |  |  |
| 20058 | 33.6 | 30.0 |  |  |  |  |
| 20059 | 34.2 | 29.5 |  |  |  |  |
| 20060 | 33.8 | 29.8 |  |  |  |  |
| 20061 | 34.3 | 30.3 |  |  |  |  |
| 20062 | 34.3 | 31.3 |  |  |  |  |
| 20063 | 34.2 | 29.8 |  |  |  |  |
| 20064 | 34.0 | 30.1 |  |  |  |  |
| 20065 | 33.5 | 29.9 |  |  |  |  |
| 20066 | 33.9 | 30.1 |  |  |  |  |
| 20067 | 33.5 | 29.8 |  |  |  |  |
| 20068 | 33.9 | 30.1 |  |  |  |  |
| 20069 | 34.0 | 30.7 |  |  |  |  |
| 20070 | 33.8 | 30.4 |  |  |  |  |
| 20071 | 34.0 | 30.2 |  |  |  |  |
| 20072 | 34.3 | 30.7 |  |  |  |  |
| 20073 | 34.1 | 30.1 |  |  |  |  |
| 20074 | 34.6 | 30.7 |  |  |  |  |
| 20075 | 34.9 | 32.0 |  |  |  |  |
| 20076 | 33.8 | 30.4 |  |  |  |  |
| 20077 | 34.1 | 30.1 |  |  |  |  |
| 20078 | 34.2 | 30.4 |  |  |  |  |
| 20079 | 33.6 | 30.2 |  |  |  |  |
| 20080 | 33.8 | 30.6 |  |  |  |  |
| 20081 | 34.7 | 33.0 |  |  |  |  |
| 20082 | 33.6 | 30.2 |  |  |  |  |
| 20083 | 34.3 | 30.3 |  |  |  |  |
| 20084 | 34.6 | 30.4 |  |  |  |  |
| 20085 | 33.4 | 30.5 |  |  |  |  |
| 20086 | 32.5 | 31.3 |  |  |  |  |
| 20087 | 32.0 | 28.4 |  |  |  |  |
| 20088 | 32.7 | 30.9 |  |  |  |  |
| 20089 | 34.7 | 31.7 |  |  |  |  |
| 20090 | 33.0 | 31.4 |  |  |  |  |
| 20091 | 33.2 | ND |  |  |  |  |
| 20092 | 32.4 | 30.5 |  |  |  |  |
| 20093 | 32.5 | 29.4 |  |  |  |  |
| 20094 | 33.2 | 29.5 |  |  |  |  |
| 20095 | 32.7 | 28.7 |  |  |  |  |
| 20096 | 33.2 | 30.5 |  |  |  |  |
| 20097 | 32.6 | 30.4 |  |  |  |  |
| 20098 | 32.7 | 30.6 |  |  |  |  |
| 20099 | 32.5 | 30.4 |  |  |  |  |
| 20100 | 32.8 | 30.4 |  |  |  |  |
| 20101 | 32.8 | 30.4 |  |  |  |  |
| 20102 | ND | ND |  |  |  |  |
| 20103 | 33.1 | ND |  |  |  |  |
| 20104 | 33.0 | ND |  |  |  |  |
| 20105 | 32.6 | 30.5 |  |  |  |  |
| 20106 | 32.2 | 29.9 |  |  |  |  |
| 20107 | 32.8 | 30.5 |  |  |  |  |
| 20108 | 33.1 | 30.3 |  |  |  |  |
| 20109 | 33.0 | 30.5 |  |  |  |  |
| 20110 | 33.2 | 30.4 |  |  |  |  |
| 20111 | 33.2 | 30.5 |  |  |  |  |
| 20112 | 33.3 | 30.9 |  |  |  |  |
| 20113 | 32.7 | 30.1 |  |  |  |  |
| 20114 | 32.2 | 30.2 |  |  |  |  |
| 20115 | 32.1 | 29.8 |  |  |  |  |
| 20116 | 32.0 | 30.2 |  |  |  |  |
| 20117 | 32.7 | 30.1 |  |  |  |  |
| 20118 | 33.2 | 30.2 |  |  |  |  |
| 20119 | 33.2 | 30.4 |  |  |  |  |
| 20120 | 33.2 | 29.4 |  |  |  |  |
| 20121 | 32.7 | 29.4 |  |  |  |  |
| 20122 | 33.0 | 29.9 |  |  |  |  |
| 20123 | 32.9 | 30.3 |  |  |  |  |
| 20124 | 32.7 | 29.6 |  |  |  |  |
| 20125 | 33.1 | 30.5 |  |  |  |  |
| 20126 | 32.7 | 30.4 |  |  |  |  |
| 20127 | 33.5 | 31.9 |  |  |  |  |
| 20128 | 32.4 | 30.3 |  |  |  |  |
| 20129 | 32.5 | 30.0 |  |  |  |  |
| 20130 | 31.8 | 29.4 |  |  |  |  |
| 20131 | 32.5 | 30.6 |  |  |  |  |
| 20132 | 33.0 | 30.4 |  |  |  |  |
| 20133 | 32.7 | 30.3 |  |  |  |  |
| 20134 | 32.7 | 30.5 |  |  |  |  |
| 20135 | 32.8 | 30.4 |  |  |  |  |
| 20136 | 32.9 | 29.9 |  |  |  |  |
| 20137 | 32.7 | 30.2 |  |  |  |  |
| 20138 | 32.8 | 29.2 |  |  |  |  |
| 20139 | 32.2 | 30.1 |  |  |  |  |
| 20140 | 33.0 | 29.7 |  |  |  |  |
| 20141 | 32.6 | 29.8 |  |  |  |  |
| 20142 | 32.7 | 28.5 |  |  |  |  |
| 20143 | 32.2 | 29.8 |  |  |  |  |
| 20144 | 32.8 | 30.0 |  |  |  |  |
| 20145 | 32.2 | 30.2 |  |  |  |  |
| 20146 | 32.4 | 30.3 |  |  |  |  |
| 20147 | 32.2 | 30.0 |  |  |  |  |
| 20148 | 32.8 | 30.1 |  |  |  |  |
| 20149 | 32.8 | 29.4 |  |  |  |  |
| 20150 | 32.8 | 29.9 |  |  |  |  |
| 20151 | 32.7 | 30.2 |  |  |  |  |
| 20152 | 32.6 | 30.5 |  |  |  |  |
| 20153 | 32.4 | 29.9 |  |  |  |  |
| 20154 | 32.7 | 30.2 |  |  |  |  |
| 20155 | 32.4 | 30.1 |  |  |  |  |
| 20156 | 32.7 | 31.3 |  |  |  |  |
| 20157 | 33.0 | 29.8 |  |  |  |  |
| 20158 | 32.2 | 29.7 |  |  |  |  |
| 20159 | 32.5 | 30.2 |  |  |  |  |
| 20160 | 33.0 | 29.8 |  |  |  |  |
| 20161 | 32.7 | 29.6 |  |  |  |  |
| 20162 | 32.8 | 30.1 |  |  |  |  |
| 20163 | 32.7 | 29.8 |  |  |  |  |
| 20164 | 32.6 | 29.8 |  |  |  |  |
| 20165 | 32.6 | 29.5 |  |  |  |  |
| 20166 | 33.1 | 29.4 |  |  |  |  |
| 20167 | 32.2 | 29.6 |  |  |  |  |
| 20168 | 32.5 | 30.4 |  |  |  |  |
| 20169 | 32.8 | 34.3 |  |  |  |  |
| 20170 | 32.2 | 29.9 |  |  |  |  |
| 20171 | 33.1 | 30.3 |  |  |  |  |
| 20172 | 33.1 | 33.7 |  |  |  |  |
| 20173 | 31.9 | 30.3 |  |  |  |  |
| 20174 | 32.5 | 33.9 |  |  |  |  |
| 20175 | 34.4 | 28.7 |  |  |  |  |
| 20176 | 35.8 | 29.9 |  |  |  |  |
| 20177 | 35.2 | 30.1 |  |  |  |  |
| 20178 | 35.4 | 29.8 |  |  |  |  |
| 20179 | 35.0 | 29.9 |  |  |  |  |
| 20180 | 34.9 | 29.7 |  |  |  |  |
| 20181 | 35.4 | 28.8 |  |  |  |  |
| 20182 | 35.5 | 29.4 |  |  |  |  |
| 20183 | 34.9 | 29.4 |  |  |  |  |
| 20184 | 34.9 | 29.4 |  |  |  |  |
| 20185 | 34.9 | 29.3 |  |  |  |  |
| 20186 | ND | ND |  |  |  |  |
| 20187 | 35.6 | 33.3 |  |  |  |  |
| 20188 | 34.7 | 29.7 |  |  |  |  |
| 20189 | 35.7 | ND |  |  |  |  |
| 20190 | 36.7 | ND |  |  |  |  |
| 20191 | 36.5 | 39.0 |  |  |  |  |
| 20192 | 36.6 | 32.7 |  |  |  |  |
| 20193 | 36.7 | ND |  |  |  |  |
| 20194 | 37.5 | 30.3 |  |  |  |  |
| 20195 | 34.6 | 29.3 |  |  |  |  |
| 20196 | 34.7 | 29.2 |  |  |  |  |
| 20197 | 36.0 | 30.6 |  |  |  |  |
| 20198 | 34.5 | 29.0 |  |  |  |  |
| 20199 | 35.4 | 29.4 |  |  |  |  |
| 20200 | 35.1 | 29.4 |  |  |  |  |
| 20201 | 35.6 | 30.1 |  |  |  |  |
| 20202 | 35.0 | 29.4 |  |  |  |  |
| 20203 | 36.0 | 32.9 |  |  |  |  |
| 20204 | 34.7 | 29.5 |  |  |  |  |
| 20205 | 35.0 | 30.3 |  |  |  |  |
| 20206 | 35.2 | 31.7 |  |  |  |  |
| 20207 | 34.6 | 30.2 |  |  |  |  |
| 20208 | 35.8 | 30.9 |  |  |  |  |
| 20209 | 35.3 | 30.4 |  |  |  |  |
| 20210 | 35.1 | 29.9 |  |  |  |  |
| 20211 | 35.4 | 30.2 |  |  |  |  |
| 20212 | 34.8 | 30.2 |  |  |  |  |
| 20213 | 34.8 | 30.2 |  |  |  |  |
| 20214 | 35.2 | 29.6 |  |  |  |  |
| 20215 | 35.2 | 29.9 |  |  |  |  |
| 20216 | 35.4 | 29.7 |  |  |  |  |
| 20217 | 35.3 | 30.4 |  |  |  |  |
| 20218 | 35.1 | 29.4 |  |  |  |  |
| 20219 | 34.8 | 29.4 |  |  |  |  |
| 20220 | 34.5 | 28.7 |  |  |  |  |
| 20221 | 34.6 | 29.9 |  |  |  |  |
| 20222 | 34.8 | 28.9 |  |  |  |  |
| 20223 | 35.1 | 29.2 |  |  |  |  |
| 20224 | 35.0 | 29.0 |  |  |  |  |
| 20225 | 35.0 | 29.3 |  |  |  |  |
| 20226 | 35.0 | 29.6 |  |  |  |  |
| 20227 | 35.3 | 29.1 |  |  |  |  |
| 20228 | 34.7 | 28.4 |  |  |  |  |
| 20229 | 35.1 | 28.0 |  |  |  |  |
| 20230 | 35.4 | 28.5 |  |  |  |  |
| 20231 | 35.5 | 28.9 |  |  |  |  |
| 20232 | 35.6 | 29.0 |  |  |  |  |
| 20233 | 36.0 | 28.8 |  |  |  |  |
| 20234 | 34.9 | 29.2 |  |  |  |  |
| 20235 | 35.2 | 29.2 |  |  |  |  |
| 20236 | ND | ND |  |  |  |  |
| 20237 | 35.3 | 28.6 |  |  |  |  |
| 20238 | 35.0 | 28.7 |  |  |  |  |
| 20239 | 34.6 | 28.7 |  |  |  |  |
| 20240 | 35.4 | 28.9 |  |  |  |  |
| 20241 | 35.6 | 29.3 |  |  |  |  |
| 20242 | 35.0 | 29.7 |  |  |  |  |
| 20243 | 35.0 | 29.8 |  |  |  |  |
| 20244 | 35.3 | 30.1 |  |  |  |  |
| 20245 | 35.2 | 33.2 |  |  |  |  |
| 20246 | 35.1 | 30.3 |  |  |  |  |
| 20247 | 35.6 | 30.3 |  |  |  |  |
| 20248 | 35.6 | 30.4 |  |  |  |  |
| 20249 | 34.7 | 30.4 |  |  |  |  |
| 20250 | 34.9 | 28.9 |  |  |  |  |
| 20251 | 36.0 | 28.5 |  |  |  |  |
| 20252 | 35.3 | 28.6 |  |  |  |  |
| 20253 | 34.9 | 29.0 |  |  |  |  |
| 20254 | 35.7 | 29.2 |  |  |  |  |
| 20255 | 35.6 | 29.2 |  |  |  |  |
| 20256 | 35.2 | 29.7 |  |  |  |  |
| 20257 | 34.4 | 28.4 |  |  |  |  |
| 20258 | 35.2 | 30.4 |  |  |  |  |
| 20259 | 35.5 | 32.4 |  |  |  |  |
| 20260 | 35.5 | 32.0 |  |  |  |  |
| 20261 | 35.4 | 31.8 |  |  |  |  |
| 20262 | 34.8 | 30.7 |  |  |  |  |
| 20263 | 35.6 | 32.0 |  |  |  |  |
| 20264 | 35.1 | 31.9 |  |  |  |  |
| 20265 | 33.5 | 28.2 |  |  |  |  |
