# Supplementary material for: Molecular detection of SARS-CoV-2 using a reagent-free approach
Source: PLoS One. 2020 Dec 7;15(12):e0243266. doi: 10.1371/journal.pone.0243266 (PMC7721139; doi:10.1371/journal.pone.0243266)
Supplement: S4 Table — (DOCX) [file pone.0243266.s004.docx]

**S4 Table.** Evaluation of the sensitivity of the heat treatment method using different commercially available RT-PCR kits (Data used in Table 3). After excluding samples with a Ct<22.0, a total of 67 samples that were initially tested positive for SARS Cov-2 on the conventional assay (NA/ABI, Original) were thawed and retested for SARS-CoV-2 following re-extraction and detection using the conventional assay (NA/ABI, post-freezing) or following heat treatment and detection using different commercially available RT-PCR buffers (see Table 3 for buffer details). Samples have been grouped by swab type and sorted in descending Ct order (original result). Only the 59 samples that were confirmed positive post-freezing (NA/ABI, post-freezing) were considered for the % positive calculations, ie 100%. Data from the columns highlighted in green and orange were collated in S6 to generate Fig 1.

|  |  |  | **Original** | **Retest (post freezing)** | | | | | |
| --- | --- | --- | --- | --- | --- | --- | --- | --- | --- |
| **Patient num** | **Swab type** | **Buffer** | **NA/ABI** | **NA/ABI** | **Heat Treament** | | | | |
|  |  |  |  |  | **ABI TaqMan** | **Meridian FAST 1-Step** | **Meridian Low LOD** | **Quantabio ToughMix** | **Promega GoTaq** |
| 007967 | Remel conical red swab | VTM | 37.4 | ND | ND | ND | ND | ND | 39.7 |
| 007971 | Remel conical red swab | VTM | 37.1 | 41.5 | 41.5 | 39.4 | 38.4 | 39.2 | 39.6 |
| 007984 | Remel conical red swab | VTM | 37.0 | 39.6 | 43.0 | 42.2 | 39.9 | ND | 39.8 |
| 008330 | Remel conical red swab | VTM | 36.2 | 40.9 | 39.3 | ND | 36.8 | ND | 40.4 |
| 007978 | Remel conical red swab | VTM | 34.7 | 37.0 | 35.2 | 36.6 | 35.4 | ND | 36.5 |
| 007977 | Remel conical red swab | VTM | 34.3 | 36.0 | 35.4 | 38.1 | 34.8 | 36.9 | 37.4 |
| 008641 | Remel conical red swab | VTM | 30.0 | 31.9 | 31.9 | 32.2 | 30.5 | 32.1 | 32.8 |
| 007976 | Remel conical red swab | VTM | 24.5 | 26.2 | 25.2 | 25.4 | 24.6 | 25.8 | 15.8 |
| 008027 | MWE sigma-Virocult | Virocult | 39.2 | ND | ND | ND | ND | ND | ND |
| 008029 | MWE sigma-Virocult | Virocult | 37.4 | 32.0 | 33.5 | 31.5 | 30.2 | 30.6 | 33.7 |
| 008226 | MWE sigma-Virocult | Virocult | 30.5 | 34.6 | 33.9 | 34.2 | 32.4 | 34.9 | 37.0 |
| 008231 | MWE sigma-Virocult | Virocult | 21.1 | 35.9 | 33.8 | 35.2 | 34.4 | 35.0 | 34.6 |
| 008104 | Dry Copan | Igepal | 40.1 | ND | ND | 40.7 | 40.3 | ND | ND |
| 008096 | Dry Copan | Igepal | 40.1 | 41.2 | 38.6 | 41.6 | 37.5 | ND | ND |
| 008095 | Dry Copan | Igepal | 39.2 | 38.9 | 36.1 | ND | ND | ND | ND |
| 008106 | Dry Copan | Igepal | 38.6 | 40.0 | ND | ND | ND | ND | 39.7 |
| 008092 | Dry Copan | Igepal | 38.1 | 41.8 | 38.2 | 38.6 | 38.2 | ND | 39.9 |
| 008094 | Dry Copan | Igepal | 37.1 | 39.0 | 38.3 | 38.1 | 35.4 | ND | ND |
| 008097 | Dry Copan | Igepal | 35.8 | 37.8 | 40.1 | 39.5 | 37.9 | ND | ND |
| 008083 | Dry Copan | Igepal | 34.0 | 39.3 | 38.7 | 38.6 | 38.4 | ND | 37.4 |
| 008090 | Dry Copan | Igepal | 33.2 | 36.7 | 36.3 | 38.7 | 35.5 | ND | 36.0 |
| 008088 | Dry Copan | Igepal | 32.1 | 33.8 | 32.0 | 35.6 | 31.7 | 34.9 | 34.7 |
| 008079 | Dry Copan | Igepal | 30.9 | 31.7 | 32.1 | 33.9 | 31.7 | 32.6 | 33.3 |
| 008359 | BD Dry purple swab | Igepal | 28.9 | 42.2 | ND | 39.5 | ND | ND | 37.5 |
| 008346 | BD Dry purple swab | Igepal | 28.1 | 38.7 | ND | ND | ND | ND | ND |
| 008348 | BD Dry purple swab | Igepal | 27.1 | 40.3 | 38.3 | ND | 37.7 | ND | ND |
| 008073 | Cepheid Xpert® viral TM | VTM | 28.6 | 30.8 | ND | 30.3 | 33.1 | 32.5 | 33.6 |
| 008023 | Cepheid Xpert® viral TM | VTM | 26.9 | 29.8 | ND | 29.9 | 31.7 | 31.7 | 33.3 |
| 008057 | Cepheid Xpert® viral TM | VTM | 25.6 | 28.4 | ND | 29.0 | 30.4 | 31.7 | 31.8 |
| 008000 | Cepheid Xpert® viral TM | VTM | 22.9 | 25.8 | ND | 26.4 | 27.5 | 27.6 | 28.7 |
| 008046 | Copan red skirted swab | UTM | 40.6 | 42.0 | ND | ND | ND | ND | ND |
| 008188 | Copan red skirted swab | UTM | 40.3 | ND | ND | ND | ND | ND | ND |
| 008199 | Copan red skirted swab | UTM | 40.3 | 40.4 | ND | ND | ND | ND | ND |
| 008217 | Copan red skirted swab | UTM | 39.7 | 39.4 | ND | ND | ND | ND | ND |
| 008604 | Copan red skirted swab | UTM | 39.3 | ND | ND | ND | ND | ND | ND |
| 008059 | Copan red skirted swab | UTM | 38.8 | 41.6 | ND | ND | ND | ND | ND |
| 008309 | Copan red skirted swab | UTM | 38.7 | ND | ND | ND | ND | ND | ND |
| 008216 | Copan red skirted swab | UTM | 38.7 | ND | ND | ND | ND | ND | ND |
| 008324 | Copan red skirted swab | UTM | 38.5 | 38.9 | ND | 40.9 | ND | ND | ND |
| 008594 | Copan red skirted swab | UTM | 38.2 | 40.3 | ND | ND | ND | ND | ND |
| 008595 | Copan red skirted swab | UTM | 38.2 | 38.0 | ND | 38.8 | ND | ND | ND |
| 008605 | Copan red skirted swab | UTM | 38.0 | ND | ND | ND | 42.4 | ND | ND |
| 008159 | Copan red skirted swab | UTM | 38.0 | 40.1 | ND | 40.6 | 42.3 | ND | ND |
| 008219 | Copan red skirted swab | UTM | 37.4 | 39.8 | ND | ND | ND | ND | ND |
| 008050 | Copan red skirted swab | UTM | 37.4 | 39.5 | ND | 40.2 | 41.4 | ND | ND |
| 008190 | Copan red skirted swab | UTM | 37.2 | 41.3 | ND | ND | ND | ND | ND |
| 008195 | Copan red skirted swab | UTM | 37.2 | 39.8 | ND | ND | ND | ND | ND |
| 008204 | Copan red skirted swab | UTM | 37.0 | 38.6 | ND | ND | 42.6 | ND | ND |
| 008314 | Copan red skirted swab | UTM | 37.0 | 38.0 | ND | 41.5 | ND | ND | ND |
| 008198 | Copan red skirted swab | UTM | 36.6 | 40.3 | ND | ND | ND | ND | 40.9 |
| 008462 | Copan red skirted swab | UTM | 36.5 | 40.1 | ND | ND | ND | ND | ND |
| 008060 | Copan red skirted swab | UTM | 36.3 | 37.7 | ND | 40.3 | ND | ND | ND |
| 008612 | Copan red skirted swab | UTM | 36.2 | 36.7 | ND | 38.7 | 42.2 | ND | ND |
| 008151 | Copan red skirted swab | UTM | 36.1 | 35.7 | ND | 38.8 | 39.3 | ND | 41.1 |
| 008464 | Copan red skirted swab | UTM | 36.0 | 39.9 | ND | ND | ND | ND | ND |
| 008184 | Copan red skirted swab | UTM | 35.2 | 36.6 | ND | 38.3 | 38.9 | ND | 35.9 |
| 008475 | Copan red skirted swab | UTM | 34.6 | 37.8 | ND | 39.7 | ND | 42.8 | ND |
| 008477 | Copan red skirted swab | UTM | 34.5 | 37.3 | ND | 37.8 | 40.7 | ND | 42.1 |
| 008303 | Copan red skirted swab | UTM | 32.4 | 35.8 | ND | 36.6 | 39.3 | 42.6 | 38.9 |
| 008460 | Copan red skirted swab | UTM | 30.4 | 34.1 | ND | 34.0 | 36.0 | 37.3 | 36.8 |
| 008206 | Copan red skirted swab | UTM | 26.9 | 29.1 | ND | 28.4 | 30.3 | 31.5 | 31.6 |
| 008062 | White cap unbranded | Unknown | 39.8 | 38.9 | ND | 37.5 | 37.9 | ND | 41.6 |
| 008066 | White cap unbranded | Unknown | 38.7 | 42.0 | ND | 41.5 | 42.9 | ND | ND |
| 008039 | White cap unbranded | Unknown | 37.7 | 38.2 | ND | 38.5 | 39.1 | 40.2 | 39.2 |
| 007997 | White cap unbranded | Unknown | 33.3 | 36.4 | ND | 37.5 | 38.4 | ND | ND |
| 008018 | White cap unbranded | Unknown | 28.3 | 30.4 | ND | 27.4 | 28.8 | 30.1 | 31.5 |
| 007996 | White cap unbranded | Unknown | 25.8 | 28.6 | ND | 26.7 | 27.6 | 29.2 | 29.0 |
|  |  |  |  |  |  |  |  |  |  |
|  | **Positives** | | 67 | **59** | 20 | **43** | 41 | 20 | 33 |
|  | **% positive** | |  | **100%** | 33.9% | **72.9%** | 69.5% | 33.9% | 55.9% |
|  | **Median Ct** | | 36.6 | **38.2** | 36.2 | **38.1** | 37.5 | 32.6 | 36.8 |
|  | **SD** | | 4.81 | **4.22** | 4.09 | **4.74** | 4.75 | 4.72 | 5.13 |
|  |  |  |  |  |  |  |  |  |  |
|  |  |  |  |  |  |  |  |  |  |
|  | **Median Ct (missed)** | |  | **38.9** |  | **40.1** |  |  |  |
|  | **SD (missed)** | |  | **0.99** |  | **0.95** |  |  |  |
