# Supplementary material for: Molecular detection of SARS-CoV-2 using a reagent-free approach
Source: PLoS One. 2020 Dec 7;15(12):e0243266. doi: 10.1371/journal.pone.0243266 (PMC7721139; doi:10.1371/journal.pone.0243266)
Supplement: S5 Table — (DOCX) [file pone.0243266.s005.docx]

**S5 Table**. Sensitivity assessment of the heat treatment protocol in diagnostics samples. Multiple diagnostics runs using non-screened samples (n=545) were performed in parallel using the reference assay (NA/ABI) and the heat treatment protocol (HT/Meridian Fast). Ct values of the samples that tested positive for SARS-CoV-2 are reported. The average (Ct) and SD of all the positive results and of the positive results that were missed were determined for each method. A sensitivity of 96.2% was calculated after removal of the samples showing signs of PCR inhibition using the AoGV internal control. Detection scoring key; N: both not detected, P: both positive, H: Positive on HT only, E: Positive on Extraction only. ND: Not Detected.

| **Sample** | **NA/ABI** | **HT/Meridian FAST** | **Detection** |  |  |  |  |  |
| --- | --- | --- | --- | --- | --- | --- | --- | --- |
| 15900 | ND | ND | N |  |  |  | **Heat-Treatment (HT)** | |
| 15901 | ND | 42.6 | H |  |  |  | Positives | Negatives |
| 15902 | ND | ND | N |  | **NA/ABI** | Positives | 65 | 13 |
| 15903 | ND | ND | N |  |  |  |  |  |
| 15904 | ND | ND | N |  |  |  |  |  |
| 15905 | ND | ND | N |  |  | Negatives | 10 | 457 |
| 15906 | ND | ND | N |  |  |  |  |  |
| 15907 | ND | ND | N |  |  |  |  |  |
| 15908 | ND | ND | N |  |  |  |  |  |
| 15909 | ND | ND | N |  |  | n samples | 545 |  |
| 15910 | ND | ND | N |  |  | Sensitivity | **96.2%** |  |
| 15911 | ND | ND | N |  |  |  |  |  |
| 15912 | ND | ND | N |  |  |  |  |  |
| 15913 | ND | ND | N |  |  |  |  |  |
| 15914 | ND | ND | N |  |  |  |  |  |
| 15915 | 31.1 | 35.1 | P |  |  |  |  |  |
| 15916 | ND | ND | N |  |  |  |  |  |
| 15917 | ND | ND | N |  |  |  |  |  |
| 15918 | ND | ND | N |  |  |  |  |  |
| 15919 | ND | ND | N |  |  |  |  |  |
| 15920 | ND | ND | N |  |  |  |  |  |
| 15921 | ND | ND | N |  |  |  |  |  |
| 15922 | 24.3 | 27.9 | P |  |  |  |  |  |
| 15923 | ND | ND | N |  |  |  |  |  |
| 15924 | 25.5 | 31.8 | P |  |  |  |  |  |
| 15925 | ND | ND | N |  |  |  |  |  |
| 15926 | 35.1 | ND | E |  |  |  |  |  |
| 15927 | ND | ND | N |  |  |  |  |  |
| 15928 | 33.6 | 37.7 | P |  |  |  |  |  |
| 15929 | 33.1 | 40.7 | P |  |  |  |  |  |
| 15930 | ND | ND | N |  |  |  |  |  |
| 15931 | 33.0 | 36.4 | P |  |  |  |  |  |
| 15932 | ND | ND | N |  |  |  |  |  |
| 15933 | ND | ND | N |  |  |  |  |  |
| 15934 | ND | ND | N |  |  |  |  |  |
| 15935 | ND | ND | N |  |  |  |  |  |
| 15936 | ND | ND | N |  |  |  |  |  |
| 15937 | ND | ND | N |  |  |  |  |  |
| 15938 | ND | ND | N |  |  |  |  |  |
| 15939 | ND | ND | N |  |  |  |  |  |
| 15940 | ND | ND | N |  |  |  |  |  |
| 15941 | 30.4 | 31.1 | P |  |  |  |  |  |
| 15942 | ND | ND | N |  |  |  |  |  |
| 15943 | 33.7 | 35.9 | P |  |  |  |  |  |
| 15944 | 29.7 | 32.5 | P |  |  |  |  |  |
| 15945 | ND | ND | N |  |  |  |  |  |
| 15946 | ND | ND | N |  |  |  |  |  |
| 15947 | ND | ND | N |  |  |  |  |  |
| 15948 | 31.1 | 33.4 | P |  |  |  |  |  |
| 15949 | 29.4 | 31.6 | P |  |  |  |  |  |
| 15950 | ND | ND | N |  |  |  |  |  |
| 15951 | ND | ND | N |  |  |  |  |  |
| 15952 | ND | ND | N |  |  |  |  |  |
| 15953 | ND | ND | N |  |  |  |  |  |
| 15954 | ND | 41.8 | H |  |  |  |  |  |
| 15955 | 37.0 | 39.4 | P |  |  |  |  |  |
| 15956 | 34.5 | 35.9 | P |  |  |  |  |  |
| 15957 | ND | ND | N |  |  |  |  |  |
| 15958 | ND | ND | N |  |  |  |  |  |
| 15959 | 39.5 | ND | E |  |  |  |  |  |
| 15960 | ND | ND | N |  |  |  |  |  |
| 15961 | ND | ND | N |  |  |  |  |  |
| 15962 | 33.3 | 34.4 | P |  |  |  |  |  |
| 15963 | ND | ND | N |  |  |  |  |  |
| 15964 | 35.2 | 34.6 | P |  |  |  |  |  |
| 15965 | 31.8 | 36.6 | P |  |  |  |  |  |
| 15966 | ND | ND | N |  |  |  |  |  |
| 15967 | ND | ND | N |  |  |  |  |  |
| 15968 | 27.7 | 29.0 | P |  |  |  |  |  |
| 15969 | 38.7 | 40.1 | P |  |  |  |  |  |
| 15970 | 39.8 | 40.6 | P |  |  |  |  |  |
| 15971 | ND | ND | N |  |  |  |  |  |
| 15972 | ND | ND | N |  |  |  |  |  |
| 15973 | 39.7 | ND | E |  |  |  |  |  |
| 15974 | ND | ND | N |  |  |  |  |  |
| 15975 | ND | ND | N |  |  |  |  |  |
| 15976 | ND | ND | N |  |  |  |  |  |
| 15977 | ND | ND | N |  |  |  |  |  |
| 15978 | ND | ND | N |  |  |  |  |  |
| 15979 | ND | ND | N |  |  |  |  |  |
| 15980 | ND | ND | N |  |  |  |  |  |
| 15981 | 39.8 | 38.6 | P |  |  |  |  |  |
| 15982 | ND | ND | N |  |  |  |  |  |
| 15983 | ND | ND | N |  |  |  |  |  |
| 15984 | ND | ND | N |  |  |  |  |  |
| 15985 | 40.2 | 39.1 | P |  |  |  |  |  |
| U | ND | ND | N |  |  |  |  |  |
| 15986 | 40.0 | 38.1 | P |  |  |  |  |  |
| 15987 | ND | ND | N |  |  |  |  |  |
| 15988 | 36.6 | 39.2 | P |  |  |  |  |  |
| 15989 | 38.9 | ND | E |  |  |  |  |  |
| 15990 | ND | ND | N |  |  |  |  |  |
| 15991 | ND | ND | N |  |  |  |  |  |
| 15992 | ND | ND | N |  |  |  |  |  |
| 15993 | ND | ND | N |  |  |  |  |  |
| 15994 | ND | ND | N |  |  |  |  |  |
| 15995 | ND | ND | N |  |  |  |  |  |
| 15996 | ND | ND | N |  |  |  |  |  |
| 15997 | ND | ND | N |  |  |  |  |  |
| 15998 | ND | ND | N |  |  |  |  |  |
| 15999 | ND | ND | N |  |  |  |  |  |
| 16000 | ND | ND | N |  |  |  |  |  |
| 16001 | ND | ND | N |  |  |  |  |  |
| 16002 | ND | ND | N |  |  |  |  |  |
| 16003 | ND | ND | N |  |  |  |  |  |
| 16004 | ND | ND | N |  |  |  |  |  |
| 16005 | ND | ND | N |  |  |  |  |  |
| 16006 | ND | ND | N |  |  |  |  |  |
| 16007 | ND | ND | N |  |  |  |  |  |
| 16008 | ND | ND | N |  |  |  |  |  |
| 16009 | ND | ND | N |  |  |  |  |  |
| 16010 | ND | ND | N |  |  |  |  |  |
| 16011 | ND | ND | N |  |  |  |  |  |
| 16012 | ND | ND | N |  |  |  |  |  |
| 16013 | ND | ND | N |  |  |  |  |  |
| 16014 | ND | ND | N |  |  |  |  |  |
| 16015 | ND | ND | N |  |  |  |  |  |
| 16016 | ND | ND | N |  |  |  |  |  |
| 16017 | ND | ND | N |  |  |  |  |  |
| 16018 | ND | ND | N |  |  |  |  |  |
| 16019 | ND | ND | N |  |  |  |  |  |
| 16020 | ND | ND | N |  |  |  |  |  |
| 16021 | ND | ND | N |  |  |  |  |  |
| 16022 | ND | 38.3 | H |  |  |  |  |  |
| 16023 | ND | ND | N |  |  |  |  |  |
| 16024 | 35.5 | 36.7 | P |  |  |  |  |  |
| 16025 | ND | ND | N |  |  |  |  |  |
| 16026 | ND | ND | N |  |  |  |  |  |
| 16027 | ND | ND | N |  |  |  |  |  |
| 16028 | ND | ND | N |  |  |  |  |  |
| 16029 | ND | ND | N |  |  |  |  |  |
| 16030 | ND | ND | N |  |  |  |  |  |
| 16031 | ND | ND | N |  |  |  |  |  |
| U | ND | ND | N |  |  |  |  |  |
| 16032 | ND | ND | N |  |  |  |  |  |
| 16033 | 26.8 | 33.6 | P |  |  |  |  |  |
| 16034 | ND | ND | N |  |  |  |  |  |
| 16035 | ND | ND | N |  |  |  |  |  |
| 16036 | ND | ND | N |  |  |  |  |  |
| 16037 | ND | ND | N |  |  |  |  |  |
| 16038 | ND | ND | N |  |  |  |  |  |
| 16039 | 24.1 | 27.2 | P |  |  |  |  |  |
| 16040 | ND | ND | N |  |  |  |  |  |
| 16041 | ND | ND | N |  |  |  |  |  |
| 16042 | ND | ND | N |  |  |  |  |  |
| 16043 | 35.9 | 39.4 | P |  |  |  |  |  |
| 16044 | 35.9 | ND | E |  |  |  |  |  |
| 16045 | ND | ND | N |  |  |  |  |  |
| 16046 | ND | ND | N |  |  |  |  |  |
| 16047 | ND | ND | N |  |  |  |  |  |
| 16048 | ND | ND | N |  |  |  |  |  |
| 16049 | ND | ND | N |  |  |  |  |  |
| 16050 | ND | ND | N |  |  |  |  |  |
| 16051 | ND | ND | N |  |  |  |  |  |
| 16052 | ND | ND | N |  |  |  |  |  |
| 16053 | ND | ND | N |  |  |  |  |  |
| 16054 | ND | ND | N |  |  |  |  |  |
| 16055 | ND | ND | N |  |  |  |  |  |
| 16056 | 35.8 | 41.3 | P |  |  |  |  |  |
| 16057 | ND | ND | N |  |  |  |  |  |
| 16058 | ND | ND | N |  |  |  |  |  |
| 16059 | ND | ND | N |  |  |  |  |  |
| 16060 | ND | ND | N |  |  |  |  |  |
| 16061 | 37.8 | ND | E |  |  |  |  |  |
| 16062 | ND | ND | N |  |  |  |  |  |
| 16063 | ND | ND | N |  |  |  |  |  |
| 16064 | 36.7 | ND | E |  |  |  |  |  |
| 16065 | ND | ND | N |  |  |  |  |  |
| 16066 | ND | 42.5 | H |  |  |  |  |  |
| 16067 | ND | ND | N |  |  |  |  |  |
| 16068 | ND | ND | N |  |  |  |  |  |
| 16069 | ND | ND | N |  |  |  |  |  |
| 16070 | ND | ND | N |  |  |  |  |  |
| 16071 | ND | ND | N |  |  |  |  |  |
| 16072 | ND | ND | N |  |  |  |  |  |
| 16073 | ND | 38.8 | H |  |  |  |  |  |
| 16074 | ND | ND | N |  |  |  |  |  |
| 16075 | 35.4 | 40.3 | P |  |  |  |  |  |
| 16076 | ND | ND | N |  |  |  |  |  |
| 16077 | ND | ND | N |  |  |  |  |  |
| 16078 | ND | ND | N |  |  |  |  |  |
| 16079 | ND | ND | N |  |  |  |  |  |
| 16080 | ND | ND | N |  |  |  |  |  |
| 16081 | ND | ND | N |  |  |  |  |  |
| 16082 | 36.3 | 39.1 | P |  |  |  |  |  |
| 16083 | ND | ND | N |  |  |  |  |  |
| 16084 | ND | ND | N |  |  |  |  |  |
| 16085 | ND | ND | N |  |  |  |  |  |
| 16086 | 37.6 | 41.7 | P |  |  |  |  |  |
| 16087 | ND | ND | N |  |  |  |  |  |
| 16088 | ND | ND | N |  |  |  |  |  |
| 16089 | ND | ND | N |  |  |  |  |  |
| U | ND | ND | N |  |  |  |  |  |
| 16090 | ND | ND | N |  |  |  |  |  |
| 16091 | ND | ND | N |  |  |  |  |  |
| 16092 | ND | ND | N |  |  |  |  |  |
| 16093 | ND | ND | N |  |  |  |  |  |
| 16094 | ND | ND | N |  |  |  |  |  |
| 16095 | ND | ND | N |  |  |  |  |  |
| 16096 | ND | ND | N |  |  |  |  |  |
| 16097 | ND | ND | N |  |  |  |  |  |
| 16098 | ND | ND | N |  |  |  |  |  |
| 16099 | ND | ND | N |  |  |  |  |  |
| 16100 | ND | ND | N |  |  |  |  |  |
| 16101 | ND | ND | N |  |  |  |  |  |
| 16102 | ND | ND | N |  |  |  |  |  |
| 16103 | ND | ND | N |  |  |  |  |  |
| 16104 | ND | ND | N |  |  |  |  |  |
| 16105 | ND | ND | N |  |  |  |  |  |
| 16106 | ND | ND | N |  |  |  |  |  |
| 16107 | ND | ND | N |  |  |  |  |  |
| 16108 | ND | ND | N |  |  |  |  |  |
| 16109 | ND | ND | N |  |  |  |  |  |
| 16110 | ND | ND | N |  |  |  |  |  |
| 16111 | ND | ND | N |  |  |  |  |  |
| 16112 | ND | ND | N |  |  |  |  |  |
| 16113 | ND | ND | N |  |  |  |  |  |
| 16114 | ND | ND | N |  |  |  |  |  |
| 16115 | ND | ND | N |  |  |  |  |  |
| 16116 | ND | ND | N |  |  |  |  |  |
| 16117 | ND | ND | N |  |  |  |  |  |
| 16118 | ND | ND | N |  |  |  |  |  |
| 16119 | ND | ND | N |  |  |  |  |  |
| 16120 | ND | ND | N |  |  |  |  |  |
| 16121 | ND | ND | N |  |  |  |  |  |
| 16122 | ND | ND | N |  |  |  |  |  |
| 16123 | ND | ND | N |  |  |  |  |  |
| 16124 | ND | ND | N |  |  |  |  |  |
| 16125 | ND | ND | N |  |  |  |  |  |
| 16126 | ND | ND | N |  |  |  |  |  |
| 16127 | ND | ND | N |  |  |  |  |  |
| 16128 | ND | ND | N |  |  |  |  |  |
| 16129 | ND | ND | N |  |  |  |  |  |
| 16130 | ND | ND | N |  |  |  |  |  |
| 16131 | ND | ND | N |  |  |  |  |  |
| 16132 | ND | ND | N |  |  |  |  |  |
| 16133 | ND | ND | N |  |  |  |  |  |
| 16134 | ND | ND | N |  |  |  |  |  |
| 16135 | ND | ND | N |  |  |  |  |  |
| 16136 | ND | ND | N |  |  |  |  |  |
| 16137 | ND | ND | N |  |  |  |  |  |
| 16138 | ND | ND | N |  |  |  |  |  |
| 16139 | ND | ND | N |  |  |  |  |  |
| 16140 | ND | ND | N |  |  |  |  |  |
| 16141 | ND | ND | N |  |  |  |  |  |
| 16142 | ND | ND | N |  |  |  |  |  |
| 16143 | ND | ND | N |  |  |  |  |  |
| 16144 | ND | ND | N |  |  |  |  |  |
| 16145 | ND | ND | N |  |  |  |  |  |
| 16146 | ND | ND | N |  |  |  |  |  |
| 16147 | ND | ND | N |  |  |  |  |  |
| 16148 | ND | ND | N |  |  |  |  |  |
| 16149 | ND | ND | N |  |  |  |  |  |
| 16150 | ND | ND | N |  |  |  |  |  |
| 16151 | ND | ND | N |  |  |  |  |  |
| 16152 | ND | ND | N |  |  |  |  |  |
| 16153 | ND | ND | N |  |  |  |  |  |
| 16154 | ND | ND | N |  |  |  |  |  |
| 16155 | ND | ND | N |  |  |  |  |  |
| 16156 | ND | ND | N |  |  |  |  |  |
| 16157 | ND | ND | N |  |  |  |  |  |
| 16158 | ND | ND | N |  |  |  |  |  |
| 16159 | ND | ND | N |  |  |  |  |  |
| 16160 | ND | ND | N |  |  |  |  |  |
| 16161 | ND | ND | N |  |  |  |  |  |
| 16162 | 33.4 | 28.3 | P |  |  |  |  |  |
| 16163 | ND | ND | N |  |  |  |  |  |
| 16164 | ND | ND | N |  |  |  |  |  |
| 16165 | ND | ND | N |  |  |  |  |  |
| 16166 | ND | ND | N |  |  |  |  |  |
| 16167 | ND | ND | N |  |  |  |  |  |
| 16168 | ND | ND | N |  |  |  |  |  |
| 16169 | ND | ND | N |  |  |  |  |  |
| 16170 | 33.8 | 27.0 | P |  |  |  |  |  |
| 16171 | ND | ND | N |  |  |  |  |  |
| 16172 | 38.5 | ND | E |  |  |  |  |  |
| 16173 | ND | ND | N |  |  |  |  |  |
| 16174 | ND | ND | N |  |  |  |  |  |
| 16175 | ND | ND | N |  |  |  |  |  |
| 16176 | ND | ND | N |  |  |  |  |  |
| U | ND | ND | N |  |  |  |  |  |
| 16177 | ND | ND | N |  |  |  |  |  |
| 16178 | ND | ND | N |  |  |  |  |  |
| 16179 | ND | ND | N |  |  |  |  |  |
| 16180 | ND | ND | N |  |  |  |  |  |
| 16181 | ND | ND | N |  |  |  |  |  |
| 16182 | ND | ND | N |  |  |  |  |  |
| 16183 | ND | ND | N |  |  |  |  |  |
| 16184 | ND | ND | N |  |  |  |  |  |
| 16185 | ND | ND | N |  |  |  |  |  |
| 16186 | ND | ND | N |  |  |  |  |  |
| 16187 | ND | ND | N |  |  |  |  |  |
| 16188 | ND | ND | N |  |  |  |  |  |
| 16189 | ND | ND | N |  |  |  |  |  |
| 16190 | ND | ND | N |  |  |  |  |  |
| 16191 | ND | ND | N |  |  |  |  |  |
| 16192 | 26.2 | 26.4 | P |  |  |  |  |  |
| 16193 | ND | ND | N |  |  |  |  |  |
| 16194 | ND | ND | N |  |  |  |  |  |
| 16195 | ND | ND | N |  |  |  |  |  |
| 16196 | ND | ND | N |  |  |  |  |  |
| 16197 | ND | ND | N |  |  |  |  |  |
| 16198 | 40.2 | 38.5 | P |  |  |  |  |  |
| 16199 | 40.2 | ND | E |  |  |  |  |  |
| 16200 | ND | ND | N |  |  |  |  |  |
| 16201 | ND | ND | N |  |  |  |  |  |
| 16202 | ND | ND | N |  |  |  |  |  |
| 16203 | 37.7 | 40.7 | P |  |  |  |  |  |
| 16204 | 38.0 | 37.7 | P |  |  |  |  |  |
| 16205 | ND | ND | N |  |  |  |  |  |
| 16206 | 32.6 | 35.8 | P |  |  |  |  |  |
| 16207 | ND | ND | N |  |  |  |  |  |
| 16208 | ND | ND | N |  |  |  |  |  |
| 16209 | ND | ND | N |  |  |  |  |  |
| 16210 | ND | ND | N |  |  |  |  |  |
| 16211 | ND | ND | N |  |  |  |  |  |
| 16212 | ND | ND | N |  |  |  |  |  |
| 16213 | 34.3 | 35.1 | P |  |  |  |  |  |
| 16214 | ND | ND | N |  |  |  |  |  |
| 16215 | ND | ND | N |  |  |  |  |  |
| 16216 | 31.0 | 30.8 | P |  |  |  |  |  |
| 16217 | ND | ND | N |  |  |  |  |  |
| 16218 | ND | 41.3 | H |  |  |  |  |  |
| 16219 | ND | ND | N |  |  |  |  |  |
| 16220 | ND | ND | N |  |  |  |  |  |
| 16221 | ND | ND | N |  |  |  |  |  |
| 16222 | ND | ND | N |  |  |  |  |  |
| 16223 | ND | ND | N |  |  |  |  |  |
| 16224 | ND | ND | N |  |  |  |  |  |
| 16225 | ND | ND | N |  |  |  |  |  |
| 16226 | ND | ND | N |  |  |  |  |  |
| 16227 | ND | ND | N |  |  |  |  |  |
| 16228 | 35.8 | 30.1 | P |  |  |  |  |  |
| 16229 | ND | ND | N |  |  |  |  |  |
| 16230 | ND | ND | N |  |  |  |  |  |
| 16231 | ND | ND | N |  |  |  |  |  |
| 16232 | ND | 43.0 | H |  |  |  |  |  |
| 16233 | ND | ND | N |  |  |  |  |  |
| 16234 | 39.0 | 39.2 | P |  |  |  |  |  |
| 16235 | ND | ND | N |  |  |  |  |  |
| 16236 | ND | ND | N |  |  |  |  |  |
| 16237 | ND | ND | N |  |  |  |  |  |
| 16238 | 31.8 | 32.9 | P |  |  |  |  |  |
| 16239 | ND | ND | N |  |  |  |  |  |
| 16240 | ND | ND | N |  |  |  |  |  |
| 16241 | 37.8 | ND | E |  |  |  |  |  |
| 16242 | ND | ND | N |  |  |  |  |  |
| 16243 | ND | ND | N |  |  |  |  |  |
| 16244 | ND | ND | N |  |  |  |  |  |
| 16245 | ND | ND | N |  |  |  |  |  |
| 16246 | ND | ND | N |  |  |  |  |  |
| 16247 | ND | ND | N |  |  |  |  |  |
| 16248 | ND | ND | N |  |  |  |  |  |
| 16249 | ND | ND | N |  |  |  |  |  |
| 16250 | ND | ND | N |  |  |  |  |  |
| 16251 | ND | ND | N |  |  |  |  |  |
| 16252 | ND | ND | N |  |  |  |  |  |
| 16253 | ND | ND | N |  |  |  |  |  |
| 16254 | ND | ND | N |  |  |  |  |  |
| 16255 | 37.6 | 40.4 | P |  |  |  |  |  |
| 16256 | 40.0 | 41.2 | P |  |  |  |  |  |
| 16257 | ND | ND | N |  |  |  |  |  |
| 16258 | ND | ND | N |  |  |  |  |  |
| 16259 | ND | ND | N |  |  |  |  |  |
| 16260 | ND | ND | N |  |  |  |  |  |
| 16261 | ND | ND | N |  |  |  |  |  |
| 16262 | ND | ND | N |  |  |  |  |  |
| 16263 | 34.4 | 37.7 | P |  |  |  |  |  |
| U | ND | ND | N |  |  |  |  |  |
| 16264 | ND | ND | N |  |  |  |  |  |
| 16265 | ND | ND | N |  |  |  |  |  |
| 16266 | ND | ND | N |  |  |  |  |  |
| 16267 | ND | ND | N |  |  |  |  |  |
| 16268 | ND | ND | N |  |  |  |  |  |
| 16269 | ND | ND | N |  |  |  |  |  |
| 16270 | ND | ND | N |  |  |  |  |  |
| 16271 | ND | ND | N |  |  |  |  |  |
| 16272 | ND | ND | N |  |  |  |  |  |
| 16273 | ND | ND | N |  |  |  |  |  |
| 16274 | ND | ND | N |  |  |  |  |  |
| 16275 | ND | ND | N |  |  |  |  |  |
| 16276 | ND | ND | N |  |  |  |  |  |
| 16277 | 22.8 | 23.1 | P |  |  |  |  |  |
| 16278 | ND | ND | N |  |  |  |  |  |
| 16279 | 37.8 | 38.2 | P |  |  |  |  |  |
| 16280 | ND | ND | N |  |  |  |  |  |
| 16281 | ND | ND | N |  |  |  |  |  |
| 16282 | ND | ND | N |  |  |  |  |  |
| 16283 | ND | ND | N |  |  |  |  |  |
| 16284 | ND | ND | N |  |  |  |  |  |
| 16285 | ND | ND | N |  |  |  |  |  |
| 16286 | ND | ND | N |  |  |  |  |  |
| 16287 | ND | ND | N |  |  |  |  |  |
| 16288 | ND | ND | N |  |  |  |  |  |
| 16289 | ND | ND | N |  |  |  |  |  |
| 16290 | ND | ND | N |  |  |  |  |  |
| 16291 | ND | ND | N |  |  |  |  |  |
| 16292 | 38.0 | 37.4 | P |  |  |  |  |  |
| 16293 | ND | ND | N |  |  |  |  |  |
| 16294 | ND | ND | N |  |  |  |  |  |
| 16295 | ND | ND | N |  |  |  |  |  |
| 16296 | ND | ND | N |  |  |  |  |  |
| 16297 | ND | ND | N |  |  |  |  |  |
| 16298 | ND | ND | N |  |  |  |  |  |
| 16299 | ND | ND | N |  |  |  |  |  |
| 16300 | ND | ND | N |  |  |  |  |  |
| 16301 | 36.9 | 35.5 | P |  |  |  |  |  |
| 16302 | ND | ND | N |  |  |  |  |  |
| 16303 | ND | ND | N |  |  |  |  |  |
| 16304 | ND | ND | N |  |  |  |  |  |
| 16305 | ND | ND | N |  |  |  |  |  |
| 16306 | ND | ND | N |  |  |  |  |  |
| 16307 | 38.8 | 37.7 | P |  |  |  |  |  |
| 16308 | ND | ND | N |  |  |  |  |  |
| 16309 | ND | ND | N |  |  |  |  |  |
| 16310 | 31.7 | 31.3 | P |  |  |  |  |  |
| 16311 | ND | ND | N |  |  |  |  |  |
| 16312 | ND | ND | N |  |  |  |  |  |
| 16313 | ND | ND | N |  |  |  |  |  |
| 16314 | ND | ND | N |  |  |  |  |  |
| 16315 | ND | ND | N |  |  |  |  |  |
| 16316 | ND | ND | N |  |  |  |  |  |
| 16317 | ND | ND | N |  |  |  |  |  |
| 16318 | ND | ND | N |  |  |  |  |  |
| 16319 | ND | ND | N |  |  |  |  |  |
| 16320 | ND | ND | N |  |  |  |  |  |
| 16321 | ND | ND | N |  |  |  |  |  |
| 16322 | ND | ND | N |  |  |  |  |  |
| 16323 | ND | ND | N |  |  |  |  |  |
| 16324 | ND | ND | N |  |  |  |  |  |
| 16325 | 36.7 | 38.3 | P |  |  |  |  |  |
| 16326 | ND | ND | N |  |  |  |  |  |
| 16327 | ND | ND | N |  |  |  |  |  |
| 16328 | ND | ND | N |  |  |  |  |  |
| 16329 | ND | ND | N |  |  |  |  |  |
| 16330 | 39.5 | ND | E |  |  |  |  |  |
| 16331 | ND | ND | N |  |  |  |  |  |
| 16332 | ND | ND | N |  |  |  |  |  |
| 16333 | ND | ND | N |  |  |  |  |  |
| 16334 | ND | ND | N |  |  |  |  |  |
| 16335 | ND | ND | N |  |  |  |  |  |
| 16336 | 38.2 | 38.7 | P |  |  |  |  |  |
| 16337 | 33.8 | 33.7 | P |  |  |  |  |  |
| 16338 | ND | 40.0 | H |  |  |  |  |  |
| 16339 | 40.3 | ND | E |  |  |  |  |  |
| 16340 | ND | ND | N |  |  |  |  |  |
| 16341 | ND | ND | N |  |  |  |  |  |
| 16342 | 31.5 | 32.8 | P |  |  |  |  |  |
| 16343 | ND | ND | N |  |  |  |  |  |
| 16344 | ND | ND | N |  |  |  |  |  |
| 16345 | ND | ND | N |  |  |  |  |  |
| 16346 | ND | ND | N |  |  |  |  |  |
| 16347 | 35.8 | 35.3 | P |  |  |  |  |  |
| 16348 | ND | ND | N |  |  |  |  |  |
| 16349 | ND | ND | N |  |  |  |  |  |
| 16350 | ND | ND | N |  |  |  |  |  |
| U | ND | ND | N |  |  |  |  |  |
| 16351 | ND | ND | N |  |  |  |  |  |
| 16352 | ND | ND | N |  |  |  |  |  |
| 16353 | ND | ND | N |  |  |  |  |  |
| 16354 | ND | ND | N |  |  |  |  |  |
| 16355 | ND | ND | N |  |  |  |  |  |
| 16356 | ND | ND | N |  |  |  |  |  |
| 16357 | ND | ND | N |  |  |  |  |  |
| 16358 | ND | ND | N |  |  |  |  |  |
| 16359 | ND | ND | N |  |  |  |  |  |
| 16360 | ND | ND | N |  |  |  |  |  |
| 16361 | 33.4 | 33.8 | P |  |  |  |  |  |
| 16362 | ND | ND | N |  |  |  |  |  |
| 16363 | ND | ND | N |  |  |  |  |  |
| 16364 | ND | ND | N |  |  |  |  |  |
| 16365 | ND | ND | N |  |  |  |  |  |
| 16366 | ND | ND | N |  |  |  |  |  |
| 16367 | ND | ND | N |  |  |  |  |  |
| 16368 | ND | ND | N |  |  |  |  |  |
| 16369 | ND | ND | N |  |  |  |  |  |
| 16370 | ND | ND | N |  |  |  |  |  |
| 16371 | ND | ND | N |  |  |  |  |  |
| 16372 | ND | ND | N |  |  |  |  |  |
| 16373 | ND | ND | N |  |  |  |  |  |
| 16374 | ND | ND | N |  |  |  |  |  |
| 16375 | ND | ND | N |  |  |  |  |  |
| 16376 | ND | ND | N |  |  |  |  |  |
| 16377 | ND | ND | N |  |  |  |  |  |
| 16378 | ND | ND | N |  |  |  |  |  |
| 16379 | ND | ND | N |  |  |  |  |  |
| 16380 | ND | ND | N |  |  |  |  |  |
| 16381 | ND | ND | N |  |  |  |  |  |
| 16382 | ND | ND | N |  |  |  |  |  |
| 16383 | ND | ND | N |  |  |  |  |  |
| 16384 | 28.3 | 29.5 | P |  |  |  |  |  |
| 16385 | ND | ND | N |  |  |  |  |  |
| 16386 | ND | ND | N |  |  |  |  |  |
| 16387 | ND | ND | N |  |  |  |  |  |
| 16388 | ND | ND | N |  |  |  |  |  |
| 16389 | 27.4 | 28.7 | P |  |  |  |  |  |
| 16390 | ND | ND | N |  |  |  |  |  |
| 16391 | ND | ND | N |  |  |  |  |  |
| 16392 | 24.4 | 24.3 | P |  |  |  |  |  |
| 16393 | ND | ND | N |  |  |  |  |  |
| 16394 | ND | ND | N |  |  |  |  |  |
| 16395 | ND | ND | N |  |  |  |  |  |
| 16396 | ND | ND | N |  |  |  |  |  |
| 16397 | 30.9 | 31.6 | P |  |  |  |  |  |
| 16398 | ND | ND | N |  |  |  |  |  |
| 16399 | 17.3 | 19.0 | P |  |  |  |  |  |
| 16400 | ND | ND | N |  |  |  |  |  |
| 16401 | ND | ND | N |  |  |  |  |  |
| 16402 | ND | ND | N |  |  |  |  |  |
| 16403 | ND | ND | N |  |  |  |  |  |
| 16404 | ND | ND | N |  |  |  |  |  |
| 16405 | ND | ND | N |  |  |  |  |  |
| 16406 | ND | ND | N |  |  |  |  |  |
| 16407 | ND | ND | N |  |  |  |  |  |
| 16408 | ND | ND | N |  |  |  |  |  |
| 16409 | ND | ND | N |  |  |  |  |  |
| 16410 | ND | ND | N |  |  |  |  |  |
| 16411 | 20.1 | 19.6 | P |  |  |  |  |  |
| 16412 | ND | ND | N |  |  |  |  |  |
| 16413 | 21.4 | 22.3 | P |  |  |  |  |  |
| 16414 | ND | 40.6 | H |  |  |  |  |  |
| 16415 | ND | ND | N |  |  |  |  |  |
| 16416 | ND | ND | N |  |  |  |  |  |
| 16417 | ND | ND | N |  |  |  |  |  |
| 16418 | ND | ND | N |  |  |  |  |  |
| 16419 | ND | ND | N |  |  |  |  |  |
| 16420 | ND | ND | N |  |  |  |  |  |
| 16421 | ND | ND | N |  |  |  |  |  |
| 16422 | ND | ND | N |  |  |  |  |  |
| 16423 | ND | ND | N |  |  |  |  |  |
| 16424 | ND | ND | N |  |  |  |  |  |
| 16425 | ND | ND | N |  |  |  |  |  |
| 16426 | ND | ND | N |  |  |  |  |  |
| 16427 | ND | ND | N |  |  |  |  |  |
| 16428 | ND | ND | N |  |  |  |  |  |
| 16429 | ND | ND | N |  |  |  |  |  |
| 16430 | ND | ND | N |  |  |  |  |  |
| 16431 | ND | ND | N |  |  |  |  |  |
| 16432 | ND | ND | N |  |  |  |  |  |
| 16433 | 40.2 | ND | E |  |  |  |  |  |
| 16434 | ND | ND | N |  |  |  |  |  |
| 16435 | ND | 40.7 | H |  |  |  |  |  |
| 16436 | ND | ND | N |  |  |  |  |  |
| 16437 | ND | ND | N |  |  |  |  |  |
| U | ND | ND | N |  |  |  |  |  |
