# Supplementary material for: Molecular detection of SARS-CoV-2 using a reagent-free approach
Source: PLoS One. 2020 Dec 7;15(12):e0243266. doi: 10.1371/journal.pone.0243266 (PMC7721139; doi:10.1371/journal.pone.0243266)
Supplement: S6 Table — (DOCX) [file pone.0243266.s006.docx]

**S6 Table**. Ct correlation between nucleic acid extraction (NA/ABI) and heat treatment (HT/Meridian Fast) methods. Positive Ct values from S4 and S5 Tables (columns highlighted in green and orange) were collected (sorted by ascending order). Median and SD values were calculated for all the positive results (All), for the paired samples (Paired) and for the samples missed by each method (Missed). The overall ΔCt in the paired samples (n = 102) was calculated. Note, the % positive does not reflect the true sensitivity of the HT assay since only the samples positive in the NA/ABI method were used in S4 Table.

| **Sample Num** | **NA/ABI** | **HT/Meridian FAST** | **ΔCt** |  |  |  | **NA/ABI** | **HT/Meridian FAST** |
| --- | --- | --- | --- | --- | --- | --- | --- | --- |
| 16411 | 20.1 | 19.6 | 0.5 |  | All | **n** | 143 | |
| 16413 | 21.4 | 22.3 | -0.9 |  |  | **Positive** | 132 | 113 |
| 16277 | 22.8 | 23.1 | -0.3 |  |  | **% positive** | 92.3% | 79.0% |
| 16039 | 24.1 | 27.2 | -3.1 |  |  | **Median** | 36.7 | 37.7 |
| 15922 | 24.3 | 27.9 | -3.6 |  |  | **SD** | 4.75 | 4.99 |
| 15924 | 25.5 | 31.8 | -6.3 |  | Paired | **n** | 102 | |
| 008000 | 25.8 | 26.4 | -0.6 |  |  | **Median** | 35.8 | 37.1 |
| 007976 | 26.2 | 25.4 | 0.8 |  |  | **SD** | 4.81 | 4.94 |
| 16192 | 26.2 | 26.4 | -0.2 |  |  | **Average ΔCt** | -0.92 | |
| 16033 | 26.8 | 33.6 | -6.8 |  | Missed | **n** | 11 | 30 |
| 15968 | 27.7 | 29.0 | -1.3 |  |  | **Median** | 40.7 | 39.8 |
| 008057 | 28.4 | 29.0 | -0.6 |  |  | **SD** | 1.52 | 1.56 |
| 007996 | 28.6 | 26.7 | 1.9 |  |  | **Range (Missed)** | 38.3 - 43.0 | 35.1 - 42.0 |
| 008206 | 29.1 | 28.4 | 0.7 |  |  |  |  |  |
| 15949 | 29.4 | 31.6 | -2.2 |  |  |  |  |  |
| 15944 | 29.7 | 32.5 | -2.8 |  |  |  |  |  |
| 008023 | 29.8 | 29.9 | -0.1 |  |  |  |  |  |
| 008018 | 30.4 | 27.4 | 3.0 |  |  |  |  |  |
| 15941 | 30.4 | 31.1 | -0.7 |  |  |  |  |  |
| 008073 | 30.8 | 30.3 | 0.5 |  |  |  |  |  |
| 16216 | 31.0 | 30.8 | 0.2 |  |  |  |  |  |
| 15948 | 31.1 | 33.4 | -2.3 |  |  |  |  |  |
| 15915 | 31.1 | 35.1 | -4.0 |  |  |  |  |  |
| 16342 | 31.5 | 32.8 | -1.3 |  |  |  |  |  |
| 008079 | 31.7 | 33.9 | -2.2 |  |  |  |  |  |
| 16310 | 31.7 | 31.3 | 0.4 |  |  |  |  |  |
| 15965 | 31.8 | 36.6 | -4.8 |  |  |  |  |  |
| 16238 | 31.8 | 32.9 | -1.1 |  |  |  |  |  |
| 008641 | 31.9 | 32.2 | -0.3 |  |  |  |  |  |
| 008029 | 32.0 | 31.5 | 0.6 |  |  |  |  |  |
| 16206 | 32.6 | 35.8 | -3.2 |  |  |  |  |  |
| 15931 | 33.0 | 36.4 | -3.4 |  |  |  |  |  |
| 15929 | 33.1 | 40.7 | -7.6 |  |  |  |  |  |
| 15962 | 33.3 | 34.4 | -1.1 |  |  |  |  |  |
| 16162 | 33.4 | 28.3 | 5.1 |  |  |  |  |  |
| 16361 | 33.4 | 33.8 | -0.4 |  |  |  |  |  |
| 15928 | 33.6 | 37.7 | -4.1 |  |  |  |  |  |
| 15943 | 33.7 | 35.9 | -2.2 |  |  |  |  |  |
| 16170 | 33.8 | 27.0 | 6.8 |  |  |  |  |  |
| 16337 | 33.8 | 33.7 | 0.1 |  |  |  |  |  |
| 008088 | 33.8 | 35.6 | -1.8 |  |  |  |  |  |
| 008460 | 34.1 | 34.0 | 0.1 |  |  |  |  |  |
| 16213 | 34.3 | 35.1 | -0.8 |  |  |  |  |  |
| 16263 | 34.4 | 37.7 | -3.3 |  |  |  |  |  |
| 15956 | 34.5 | 35.9 | -1.4 |  |  |  |  |  |
| 008226 | 34.6 | 34.2 | 0.4 |  |  |  |  |  |
| 15964 | 35.2 | 34.6 | 0.6 |  |  |  |  |  |
| 16075 | 35.4 | 40.3 | -4.9 |  |  |  |  |  |
| 16024 | 35.5 | 36.7 | -1.2 |  |  |  |  |  |
| 008151 | 35.7 | 38.8 | -3.1 |  |  |  |  |  |
| 008303 | 35.8 | 36.6 | -0.9 |  |  |  |  |  |
| 16228 | 35.8 | 30.1 | 5.7 |  |  |  |  |  |
| 16347 | 35.8 | 35.3 | 0.5 |  |  |  |  |  |
| 16056 | 35.8 | 41.3 | -5.5 |  |  |  |  |  |
| 008231 | 35.9 | 35.2 | 0.7 |  |  |  |  |  |
| 16043 | 35.9 | 39.4 | -3.5 |  |  |  |  |  |
| 007977 | 36.0 | 38.1 | -2.1 |  |  |  |  |  |
| 16082 | 36.3 | 39.1 | -2.8 |  |  |  |  |  |
| 007997 | 36.4 | 37.5 | -1.1 |  |  |  |  |  |
| 15988 | 36.6 | 39.2 | -2.6 |  |  |  |  |  |
| 008184 | 36.6 | 38.3 | -1.7 |  |  |  |  |  |
| 008612 | 36.7 | 38.7 | -2.1 |  |  |  |  |  |
| 008090 | 36.7 | 38.7 | -2.0 |  |  |  |  |  |
| 16325 | 36.7 | 38.3 | -1.6 |  |  |  |  |  |
| 16301 | 36.9 | 35.5 | 1.4 |  |  |  |  |  |
| 007978 | 37.0 | 36.6 | 0.4 |  |  |  |  |  |
| 15955 | 37.0 | 39.4 | -2.4 |  |  |  |  |  |
| 008477 | 37.3 | 37.8 | -0.6 |  |  |  |  |  |
| 16255 | 37.6 | 40.4 | -2.8 |  |  |  |  |  |
| 16086 | 37.6 | 41.7 | -4.1 |  |  |  |  |  |
| 008060 | 37.7 | 40.3 | -2.7 |  |  |  |  |  |
| 16203 | 37.7 | 40.7 | -3.0 |  |  |  |  |  |
| 16279 | 37.8 | 38.2 | -0.4 |  |  |  |  |  |
| 008475 | 37.8 | 39.7 | -1.9 |  |  |  |  |  |
| 008097 | 37.8 | 39.5 | -1.6 |  |  |  |  |  |
| 008595 | 38.0 | 38.8 | -0.8 |  |  |  |  |  |
| 008314 | 38.0 | 41.5 | -3.5 |  |  |  |  |  |
| 16292 | 38.0 | 37.4 | 0.6 |  |  |  |  |  |
| 16204 | 38.0 | 37.7 | 0.3 |  |  |  |  |  |
| 008039 | 38.2 | 38.5 | -0.3 |  |  |  |  |  |
| 16336 | 38.2 | 38.7 | -0.5 |  |  |  |  |  |
| 15969 | 38.7 | 40.1 | -1.4 |  |  |  |  |  |
| 16307 | 38.8 | 37.7 | 1.1 |  |  |  |  |  |
| 008324 | 38.9 | 40.9 | -2.0 |  |  |  |  |  |
| 008062 | 38.9 | 37.5 | 1.4 |  |  |  |  |  |
| 16234 | 39.0 | 39.2 | -0.2 |  |  |  |  |  |
| 008094 | 39.0 | 38.1 | 1.0 |  |  |  |  |  |
| 008083 | 39.3 | 38.6 | 0.7 |  |  |  |  |  |
| 008050 | 39.5 | 40.2 | -0.8 |  |  |  |  |  |
| 007984 | 39.6 | 42.2 | -2.6 |  |  |  |  |  |
| 15981 | 39.8 | 38.6 | 1.2 |  |  |  |  |  |
| 15970 | 39.8 | 40.6 | -0.8 |  |  |  |  |  |
| 15986 | 40.0 | 38.1 | 1.9 |  |  |  |  |  |
| 16256 | 40.0 | 41.2 | -1.2 |  |  |  |  |  |
| 008159 | 40.1 | 40.6 | -0.5 |  |  |  |  |  |
| 16198 | 40.2 | 38.5 | 1.7 |  |  |  |  |  |
| 15985 | 40.2 | 39.1 | 1.1 |  |  |  |  |  |
| 008096 | 41.2 | 41.6 | -0.4 |  |  |  |  |  |
| 007971 | 41.5 | 39.4 | 2.1 |  |  |  |  |  |
| 008092 | 41.8 | 38.6 | 3.2 |  |  |  |  |  |
| 008066 | 42.0 | 41.5 | 0.6 |  |  |  |  |  |
| 008359 | 42.2 | 39.5 | 2.6 |  |  |  |  |  |
| 15926 | 35.1 | ND |  |  |  |  |  |  |
| 16044 | 35.9 | ND |  |  |  |  |  |  |
| 16064 | 36.7 | ND |  |  |  |  |  |  |
| 16061 | 37.8 | ND |  |  |  |  |  |  |
| 16241 | 37.8 | ND |  |  |  |  |  |  |
| 16172 | 38.5 | ND |  |  |  |  |  |  |
| 008204 | 38.6 | ND |  |  |  |  |  |  |
| 008346 | 38.7 | ND |  |  |  |  |  |  |
| 15989 | 38.9 | ND |  |  |  |  |  |  |
| 008095 | 38.9 | ND |  |  |  |  |  |  |
| 008217 | 39.4 | ND |  |  |  |  |  |  |
| 15959 | 39.5 | ND |  |  |  |  |  |  |
| 16330 | 39.5 | ND |  |  |  |  |  |  |
| 15973 | 39.7 | ND |  |  |  |  |  |  |
| 008195 | 39.8 | ND |  |  |  |  |  |  |
| 008219 | 39.8 | ND |  |  |  |  |  |  |
| 008464 | 39.9 | ND |  |  |  |  |  |  |
| 008106 | 40.0 | ND |  |  |  |  |  |  |
| 008462 | 40.1 | ND |  |  |  |  |  |  |
| 16199 | 40.2 | ND |  |  |  |  |  |  |
| 16433 | 40.2 | ND |  |  |  |  |  |  |
| 008594 | 40.3 | ND |  |  |  |  |  |  |
| 008348 | 40.3 | ND |  |  |  |  |  |  |
| 16339 | 40.3 | ND |  |  |  |  |  |  |
| 008198 | 40.3 | ND |  |  |  |  |  |  |
| 008199 | 40.4 | ND |  |  |  |  |  |  |
| 008330 | 40.9 | ND |  |  |  |  |  |  |
| 008190 | 41.3 | ND |  |  |  |  |  |  |
| 008059 | 41.6 | ND |  |  |  |  |  |  |
| 008046 | 42.0 | ND |  |  |  |  |  |  |
| 16022 | ND | 38.3 |  |  |  |  |  |  |
| 16073 | ND | 38.8 |  |  |  |  |  |  |
| 16338 | ND | 40.0 |  |  |  |  |  |  |
| 16414 | ND | 40.6 |  |  |  |  |  |  |
| 008104 | ND | 40.7 |  |  |  |  |  |  |
| 16435 | ND | 40.7 |  |  |  |  |  |  |
| 16218 | ND | 41.3 |  |  |  |  |  |  |
| 15954 | ND | 41.8 |  |  |  |  |  |  |
| 16066 | ND | 42.5 |  |  |  |  |  |  |
| 15901 | ND | 42.6 |  |  |  |  |  |  |
| 16232 | ND | 43.0 |  |  |  |  |  |  |
