# Supplementary material for: Molecular detection of SARS-CoV-2 using a reagent-free approach
Source: PLoS One. 2020 Dec 7;15(12):e0243266. doi: 10.1371/journal.pone.0243266 (PMC7721139; doi:10.1371/journal.pone.0243266)
Supplement: S1 Fig — (DOCX) [file pone.0243266.s007.docx]

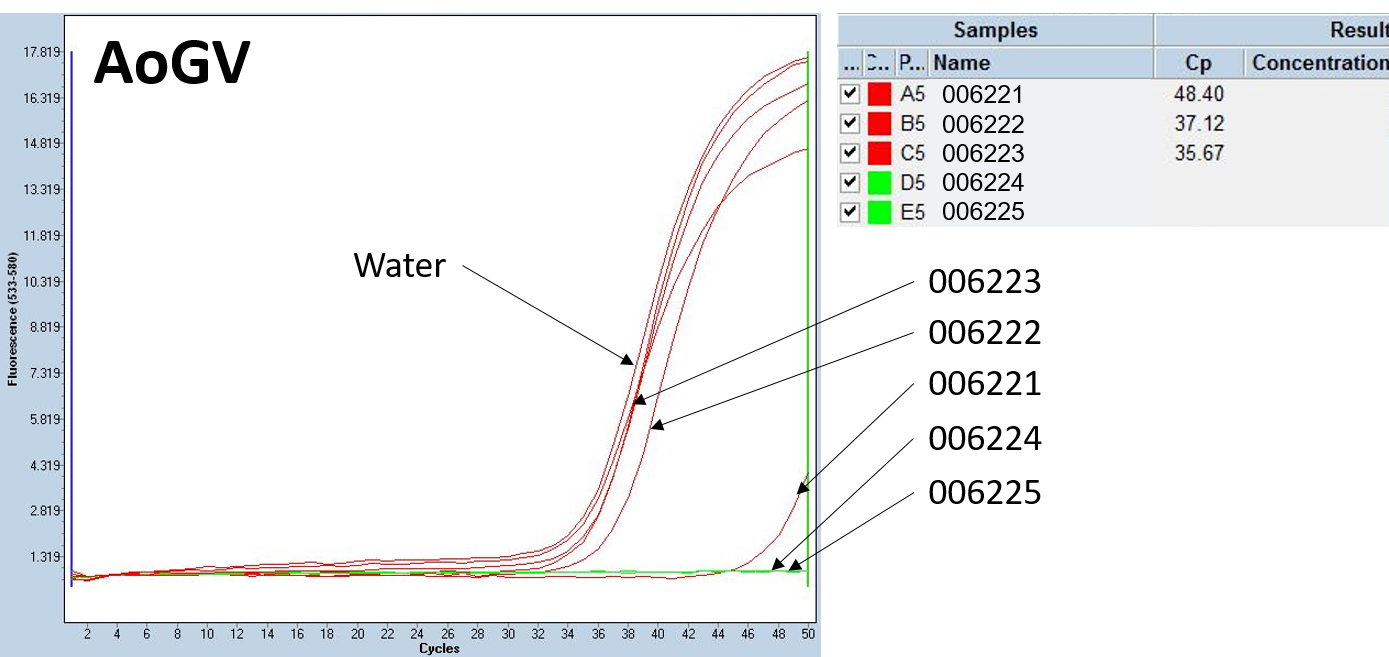


**S1 Fig.** Evidence for PCR inhibition in NPA samples. The 5 NPA samples tested in table 2 were spiked with extracted baculovirus (AoGV) DNA and tested for the presence of AoGV in a TaqMan assay. Samples 006223, 006224 and 006225 originally tested positive for SARS-CoV-2 using our conventional protocol with Ct of 33.3, 39.7 and 30.8, respectively. SARS-CoV-2 RNA was not detected in samples 006221 and 006222. “Water”: blank used to determine 100% AoGV amplification (no inhibition, Ct: 35.0).
