# Supplementary material for: Molecular detection of SARS-CoV-2 using a reagent-free approach
Source: PLoS One. 2020 Dec 7;15(12):e0243266. doi: 10.1371/journal.pone.0243266 (PMC7721139; doi:10.1371/journal.pone.0243266)
Supplement: Attachment — Submitted filename: COVID19-PLOS One Rebuttal letter RMC.docx [file pone.0243266.s008.docx]

Rebuttal letter

We thank the academic editor for his comments on our manuscript.

Please find below our responses to each point raised by the academic editor. We hope that we satisfyingly addressed all of them, and that the manuscript will be now suitable for publication.

Sincerely,

On behalf of all authors,

Ronan Calvez.

**Academic editor:**

Overall the manuscript reporting the monitoring of COV2 RNA by RTqPCR is simple and clear.

The major critiques are as follows:

**1. Detection of Cov2 RNA by RTqPCR does not mean that the virus is infectious (introduction section ie ref 7) . As for other reports, it seems to me that the monitoring of infectious virus should be provided in parallel.**

The aim of the methodology described in this paper was primarily to perform a rapid diagnostic testing for the detection of SARS-CoV-2 RNA (less than 24 h turnaround time), particularly in scenarios like hospitals with patients and staff. The paper did not intend to discriminate between infectious and non-infectious virus.

**2. Using such RTqPCR monitoring, one should be able to provide the true viral loads (as it stands for HIV infection), that most probably differ from one infected person to another one. Although these data are important from a medical perspective, they are not provided as a whole (l 169)**

The SARS-CoV-2 detection methodology described here aimed to primarily discriminate qualitatively between the presence or absence of SARS-CoV-2 RNA in patient samples. This assay has been calibrated by ddPCR to assess the low limit of detection and quantitative values could indeed be provided if the sample was quantifiable in nature (eg whole blood, plasma…). However, all the samples tested using our methodology were swab samples which are not quantifiable in nature since the buffer volume used for resuspension was variable. In addition, for an accurate result to be given, all swab samples should be taken in the exact same way (same surface area swabbed) which is unrealistic given that the samples used in this study originated from over 200 NHS Trusts in the UK.

To answer your very relevant comment regarding the clinical relevance of the amount of virus detected, each SARS-CoV-2-positive sample was reported back to the requesting authority with a caveat stating, “strongly positive” (Ct<20) or “weakly positive” (Ct>39) according to the Ct value recorded.

Two sentences have been added l 194-200 to clarify this point:

“*Since all the samples tested using this methodology were swab samples, Ct values, rather than quantitative values, were provided as this sample type does not allow a consistent and reliable viral load estimation (variability in resuspension volume and inconsistency in surface area swabbed). For clinical purposes, samples containing a Ct value greater than 39 were considered “weakly positive”, whereas samples with a Ct value less than 20 were considered “strongly positive*”.

**3. the buffer conditions (see lines 134-1554) should be available so as to optimize detection and to understand why several kits failed to detect COV2 RNA (table 2)(table 3).**

All buffers were used according to manufacturer’s instructions. The components of those buffers however were not available from the suppliers. We did request this information to understand the nature of the failed reactions but they were not able to provide it since they were protected by intellectual property or patents.

**4. RT inhibitors can be easily detected using recombinant RT enzyme in in vitro assays**

We have also investigated another RNA virus internal control derived from potato virus Y (PVY) to assess the presence of RT-specific PCR inhibitors. This assay is currently being validated as an internal control consisting of PVY RNA spiked in the RT-PCR mix, which will enable us to verify the presence of RT-inhibitors within the sample. A sentence has been added to clarify this point (l 168-169):

“*To assess the presence of RT-only inhibitors, another internal control derived from the potato virus Y (PVY) was used in parallel*.”
