# Supplementary material for: Molecular detection of SARS-CoV-2 using a reagent-free approach
Source: PLoS One. 2020 Dec 7;15(12):e0243266. doi: 10.1371/journal.pone.0243266 (PMC7721139; doi:10.1371/journal.pone.0243266)
Supplement: Attachment — Submitted filename: Response to Reviewers.docx [file pone.0243266.s009.docx]

Rebuttal letter

We thank the academic editor and reviewer for their comments on our manuscript.

Please find below our responses to each point raised by the reviewer. We hope that we satisfyingly addressed all of them, and that the manuscript will be now suitable for publication.

Sincerely,

On behalf of all authors,

Ronan Calvez.

**Reviewer #1:**

**I share the Editor’s opinion. This work is valuable owing the crucial need to have reliable and fast detection assays of SARS-Cov2 in respiratory samples. The revised version adequately addresses most of the Editor’s questions.**

**However, I think that the authors have everything in hands to build a ready to use detailed protocol that could contribute to standardization of quick testing of clinical respiratory (and possibly other types after validation) samples for their contents in RNA virus including SARS-CoV2. The assay would include the systematic addition of a known RNA probe (potato virus Y (PVY) ) to detect any RT and PCR inhibitors in heated samples. Note that the use of a DNA probe (baculovirus AoGV) maybe dispensable unless one wants to know which of the RT or DNA pol is inhibited. In this case, the author should provide how these two probes can be obtained, how to store them and if they are of any biological threat. The author can go to doi: 10.1371/journal.pone.0172358. eCollection 2017 as an example of how to write such protocol.**

**Should the authors choose not to value their work as a standard protocol, they should at least provide all the information enabling reproduction of their data including:**

**- Primers and PCR programme to quantified the AoGV DNA**

**- Primers and RT-PCR programme to quantify PVY RNA**

**- Results obtained on PVY probe detection after addition in UTM to determine why the ABI RT-qPCR kit does not work for RNA samples while it works with the AoGV DNA probe.**

The purpose of the present study was to highlight the major pitfalls when designing a heat treatment assay to replace a standard nucleic extraction approach, such as the choice of the RT-PCR mix (depending on the samples to be tested) and the design of an internal control. At the time of submission, our protocol was not fully validated since we didn’t have the PVY assay available (we could not get hold of the PVY synthetic RNA until July 2020). This is the reason why we initially used our current DNA internal control (AoGV). The data presented here was therefore analysed using a SARS-CoV-2/AoGV assay which is why we don’t find it pertinent to provide a detailed protocol, since this was not the finalised and validated version of our protocol.

As advised, we have however added the oligo sequences of all primer/probes used in this study (Table S1), details on the preparation of the main RT-PCR mix used (Table S2) and data comparing the DNA and RNA internal controls (Table S4). Cycling conditions are detailed in the materials and methods section.

Finally, to answer the last comment regarding the UTM buffer and RT inhibition in the ABI buffer, we have clarified, in the text, our hypothesis that the MMLV RT used in the ABI kit is more sensitive to inhibitors present in the UTM buffer than the RT used in the Meridian kit (lines 219-222). We didn’t get any information on the modifications brought to the RT by each company.

**Minor:**

**- the reference list needs upgrading: for example, ref 7 is now published in The New England Journal of Medicine doi: 10.1056/NEJMc2004973**

All peer-reviewd references have now been updated.

**- numbers in tables (both in main text and supplement) should be limited to only 3 significant figures (i.e. write 33.9 instead of the (statistically) meaningless 33.91)**

All numbers in manuscript, tables and supplementary data have been modified to 3 significant figures.

**- Table 1: line “patient num” the cases should be left empty (or fused with the upper ones) and not filled with “ND” since the line is solely used as headings.**

This has been amended accordingly.

**- Table S3: the mean Ct missed should be completed with the corresponding SD value. Mean, SD, and range values of each group would gained to be also mentioned in the text since pairs of data obtained with heating prep and in house NA purification protocol look statistically not significant (statistical analysis should be performed)**

Due to the additional data added, Table S3 has now become Table S6. A new table (S7) has been added. All the SARS-CoV-2-positive results from Tables S5 and S6 have been collated into this new table. The Ct missed, SD and range have been calculated from data in Table 7. All these values are now referred to in the text (lines 281-282 and 294-295).

Statistical analysis was performed to show that the Ct values measured in the HT protocol were significantly later (+0.92 cycles, line 292) than the NA method. Fig 1 was amended accordingly to reflect this.

**- Line 197: The Ct range “positive” (20**

The Ct range of positive has been clarified (20.1 to 38.9) (line 120).

The manuscript has been revised to improve fluidity. The materials and methods section has also been altered and subdivided into sub sections for clarity. More supplementary data was added to answer the reviewer’s comments (sequence information, mix preparation and AoGV vs PVY comparison). Fig 1 has also been redesigned to show statistical significance.
